# Prevalence of focal incidental breast uptake on FDG-PET/CT and risk of malignancy: a systematic review and meta-analysis

**DOI:** 10.1186/s41824-019-0063-5

**Published:** 2019-09-30

**Authors:** Else Marie Aarstad, Petter Nordhaug, Mohammad Naghavi-Behzad, Lisbet Brønsro Larsen, Oke Gerke, Malene Grubbe Hildebrandt

**Affiliations:** 1 Department of Clinical Research, University of Southern Denmark, Odense, Denmark; 2 Department of Nuclear Medicine, Odense University Hospital, Odense, Denmark; 3 Student Research Committee, Tabriz University of Medical Sciences, Tabriz, Iran; 4 Department of Radiology, Odense University Hospital, Odense, Denmark; 5 Centre for Innovative Medical Technology, Odense University Hospital, Odense, Denmark

**Keywords:** Breast cancer, FDG uptake, PET scan, Malignancy

## Abstract

**Background:**

FDG-PET/CT is increasingly used for oncologic and inflammatory diseases. Focal incidental FDG uptake occurs rarely in breast tissue but has often significant consequences. This study aimed to systematically review literature regarding focal incidental breast uptake (FIBU) on FDG-PET/CT in order to yield an update on the prevalence and risk of malignancy for FIBU.

**Methods:**

A systematic search for relevant articles published between 2012 and 2018 was performed through MEDLINE, Embase, and Cochrane databases. Studies addressing the detection of FIBU in patients without a previous history of breast malignancy were included. The QUADAS-2 was used for quality assessment, and eligible data were pooled using a fixed-effects model. *I*^2^ was calculated for the heterogeneity between studies.

**Results:**

Eight studies containing 180,002 scans were included in the systematic review. The median prevalence of FIBU for both genders was 0.52% (range 0.18–22.5%). Prevalence for women was mentioned separately in five studies and varied from 0.51 to 23.5%. One study reporting a high prevalence was based on patients being staged for known malignancy, and the word “breast” was used in the search, which may have caused selection bias. Data from four studies were eligible for meta-analysis. A high degree of heterogeneity was observed for prevalence data (*I*^2^ of 97.5%), while moderate heterogeneity was observed for data on malignancy risk assessment (*I*^2^ of 62.8%). The pooled prevalence of FIBU in women was 0.61% (range 0.56–0.66%), and the pooled prevalence of malignancy of FIBUs was 38.7% (range 34.4–43.0%). The most commonly detected malignancy was invasive ductal carcinoma.

**Conclusion:**

FIBU occurs rarely on FDG-PET/CT for female patients but yields a high risk of malignancy according to the results of published papers. Therefore, it should be considered relevant to further elucidate patients with incidentally detected FDG uptake in breast in clinical practice.

**Electronic supplementary material:**

The online version of this article (10.1186/s41824-019-0063-5) contains supplementary material, which is available to authorized users.

## Background

Molecular imaging modalities such as positron emission tomography (PET) combined with computed tomography (PET/CT) is increasingly used to improve decision-making in malignancies related to diagnosis, staging/restaging, recurrence detection, treatment planning, and response evaluation as well as prognosis (Nomura et al. 2015; Chen et al. 2018). An increasing number of clinical indications for PET/CT have been established in the past decades, mostly using the glucose analog fluorine-18-fluorodeoxyglucose (FDG) as a PET tracer (Nomura et al. 2015). FDG uptake itself is not tumor-specific, and increased FDG uptake can be seen in malignant as well as benign inflammatory lesions (Dong et al. 2016).

Incidentally detected FDG uptake has been reported in 6.7–12% of all FDG-PET/CT scans and should be considered important, since it may represent yet unrecognized malignancy. However, precise interpretation of incidental focal uptake is of high clinical and patient-relevant importance for the decision of subsequent further investigations. This requires knowledge of differential diagnosis as well as probability of malignancy for the incidentally detected FDG uptake (Pencharz et al. 2018).

The prevalence of incidentally detected primary malignancies diagnosed by FDG-PET/CT may be considerable especially for high prevalent cancers and has been reported to be from 1.0 to 1.8% (Shin et al. 2015) for lesions located at colorectal, thyroid, parotid glands, prostate, and breast (Pencharz et al. 2018; Valente 2018; Brown et al. 2015; Treglia et al. 2015). Breast cancer has claimed more than half a million female lives and over 1.7 million new cases had been diagnosed in 2016 according to reports of the Global Burden of Disease (Sharma 2019). Since the obvious gender difference in the incidence of breast cancer and the fact of being the most common malignancy among women worldwide, focal incidental breast uptake (FIBU) should be considered relevant only for women undergoing FDG-PET/CT.

The prevalence of FIBU on FDG-PET/CT and its risk of malignancy was addressed in a systematic review conducted in 2013. This review reported that FIBU was detected in 0.36–1.84% of scans performed in female patients and yielded a risk of malignancy ranging from 17.5 to 83.3% among whole evaluated patients (Bertagna et al. 2014). It is worth to have updated and precise information on FIBU and the risk of malignancy due to technological advancement and expanding usage of FDG-PET/CT in diagnostic protocols and clinical investigations (Minamimoto et al. 2015).

The aim of the current study was to systematically summarize recent published studies and to perform pooled data analysis of prevalence and risk of malignancy in patients with FIBU detected on FDG-PET/CT. In the meta-analysis part, we aimed to focus on the malignancy rate of detected FIBU in women, especially in asymptomatic patients without a known history of breast malignancy.

## Main text

### Materials and methods

This systematic review and meta-analysis was conducted at the Department of Nuclear Medicine, Odense University Hospital (OUH, Odense, Denmark), in 2018 following guidelines of PRISMA statement (Moher et al. 2009). Quality assessment was done for selected studies, and the meta-analysis was performed for eligible data.

### Search strategy

A search protocol was defined to find relevant published articles on FIBU and related malignancies discovered incidentally on FDG-PET/CT in women without a previous history of breast cancer.

Databases consisted of MEDLINE/PubMed, Scopus, and Cochrane Library were selected. Only original human studies were included, considering 2012–2018 as their publication date since previous systematic review (Bertagna et al. 2014) contained the data until July 2013. The search protocol was initiated on 21 March 2018 and was restricted to articles in English only. In July 2019, databases were checked for relevant publications for the last time.

The search strategy was based on terms and variations, it was expanded and modified for each database and consisted of three arms: (1) PET (positron emission tomography* or PET* or petscan* or FDG-PET* or PET-FDG* or PETFDG* or FDGPET* or 18F), (2) Incidental uptake (incident* or unexpect* or foc* or added or finding* or uptake or coincident*), and (3) Breast (Breast* or mamma* or mammary gland*). Regarding the MEDLINE database, search results were updated using MESH terms and free text words. Also, the previous systematic review (Bertagna et al. 2014) was considered as external validation for our literature search. The search terms are presented in Additional file 1.

### Study selection and data extraction process

The search results from databases were compiled using EndNote software (Package for Windows version 7.2). After the deletion of duplicate articles, titles and abstracts of remaining studies were examined. Manuscripts reporting prevalence or results about incidental or unexpected FDG uptake in breast through data from FDG-PET/CT scans in female patients or mix genders were included.

Exclusion criteria were considered using several modalities rather than only FDG-PET/CT, considering patients with a known history of breast cancer or only male gender, investigating other breast diseases, patient groups with cancer of unknown origin, and overlap in patients’ data (the most complete set was included). Furthermore, letters, editorials, commentaries, perspectives, reviews, case studies, and conference abstracts were excluded.

Only studies which contained data on female patients who underwent FDG-PET/CT for a reason other than breast cancer with subsequent evaluation of FIBU and risk of malignancy were included in the meta-analysis.

**Fig. 1:**
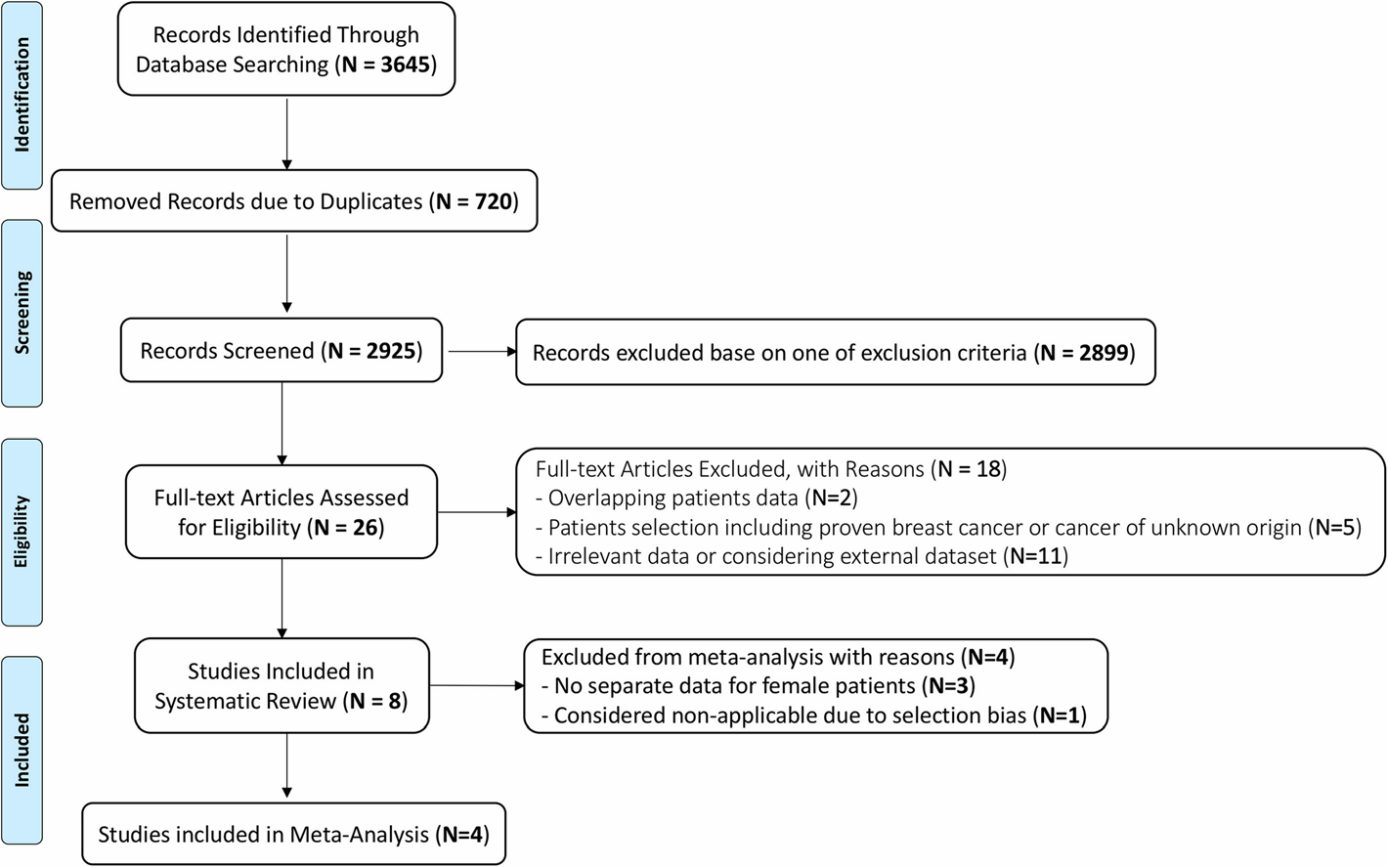
Flowchart of the search process and study selection

After an initial assessment of titles and abstracts, full-text articles were collected and evaluated for eligibility, and qualified manuscripts were selected to enter the study analysis (Fig. 1).

Scientometric information, study design, time-frame of screening, number of patients and FDG-PET/CT scans, number of patients undergoing further investigation, lost to follow-up, histopathology, and rate of malignancies among detected lesions were extracted. If needed, authors of published studies were contacted to obtain additional information. All steps were conducted by two researchers (EMA and PN) independently, resolving any outstanding disagreement by supervision of senior researcher (MGH). The QUADAS-2 tool was used to assess the methodological quality of selected studies (Whiting and Sterne 2012). FDG-PET/CT was the index test and the histopathology result was used as a reference standard for all malignant lesions, while histopathology results or at least 6 months’ imaging follow-up were applied for benign lesions. The studies assessments were done in categories of low risk, high risk, and unclear risk in all parameters, and the studies with high-quality assessment were entered to the meta-analysis. Also, for the meta-analysis part, the studies with significant bias were excluded from the final analysis.

## Statistical analysis

The prevalence of FIBU and risk of malignancy reflected by the prevalence of primary breast cancer were calculated through the median and standard deviation for each of the included studies for both genders. Meta-analysis was performed using fixed-effects model and Mantel and Haenszel method. A forest plot was derived to graphically display the point estimate considering 95% confidence interval (CI) per-study basis and to display the summary estimate for prevalence (incl. its 95% CI) across the studies (Egger et al. 1997; Sterne and Harbord 2004; Sterne et al. 2001; Sterne and Egger 2001). Also, the weight of the different studies is proportionally represented by the size of the squares and given in percentage on the right-hand side of the forest plot. A funnel plot was derived to assess publication bias visually for each analysis, and the heterogeneity of the studies was measured using *I*^2^ value (Higgins et al. 2003). All analyses were done by using STATA/MP 15.0 (StataCorp, College Station, TX).

## Results

### Literature search and study selection

The initial search resulted in 3645 records as shown in Fig. 1. Twenty-six articles were considered for full-text assessment after removing duplications and evaluation of eligibility for inclusion. Eighteen studies were excluded due to different reasons, and eight studies (Shin et al. 2015; Minamimoto et al. 2015; Bertagna et al. 2015; Chae et al. 2012; Kim et al. 2012; Benveniste et al. 2015; Dunne et al. 2013; Lim et al. 2013) were included in the systematic review.

### Quality assessment

**Fig. 2:**
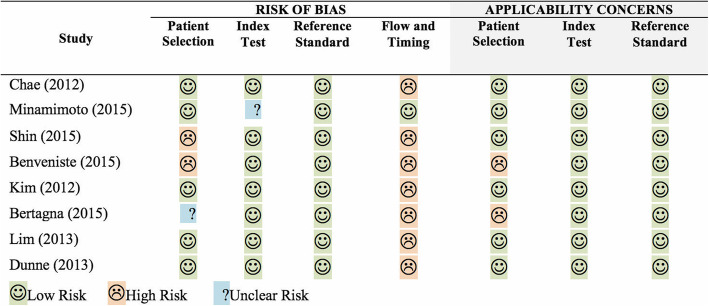
Methodological quality assessment of included studies using QUADAS-2 tool

Individual assessment of the quality and risk of bias for each of the studies using QUADAS-2 tool can be seen in Fig. 2.

Risk of bias was assessed low regarding index test (seven studies) and reference standard (eight studies). Patient selection was assessed with low risk of bias in five studies (Minamimoto et al. 2015; Chae et al. 2012; Kim et al. 2012; Dunne et al. 2013; Lim et al. 2013), high risk in two (Shin et al. 2015; Benveniste et al. 2015), and unclear in one study (Bertagna et al. 2015). In the flow and time domain sections, seven of the selected studies were assessed with high risk of bias which could be explained by a high number of patients lost to follow-up in almost all of the studies. Regarding applicability of index test and reference standard, all of the studies were assessed as low risk, and regarding patient selection, two studies (Bertagna et al. 2015; Benveniste et al. 2015) were considered at high risk while another six studies were assessed at low risk of bias.

### Study and patient characteristics

Eight studies with retrospective study designs published from 2012 to 2015 were included in the systematic review. The patients had FDG-PET/CT scans performed from April 2004 to October 2013. An overview of the included studies is seen in Table 1. Included studies provided a total of 180,002 scans of which 95,457 (53%) scans were performed in female patients.

**Table 1:** Characteristics of included studies and prevalence of FIBU

Items	Year	Country	Time period	Source of population	No. of scans		Detected FIBU^*^	
Studies					Female	Total	Female	Total
Chae et al. (2012)	2012	Korea	2005–2010	Patients with known or suspected malignancy or screening for malignancy	NR	32,988	NR	131 (0.40)
Kim et al. (2012)	2012	Korea	2004–2010	Patients underwent PET for staging of known primary malignancies or cancer screenings	NR	5214	NR	27 (0.52)
Dunne et al. (2013)	2013	Ireland	2009–2012	Patients underwent PET for any reason reporting breast abnormality	2719	6050	50 (1.84)	50 (0.83)
Lim et al. (2013)	2013	Korea	2006–2011	Patients with known malignancy or asymptomatic women for their health check-up	7594		43 (0.57)	
Minamimoto et al. (2015)	2015	Japan	2006–2009	Nationwide survey of the FDG-PET cancer screening program	62,054		317 (0.51)	
Benveniste et al. (2015)	2015	USA	2005–2011	Patients underwent PET for any reason including the word “breast” in their report	1866	1951	438 (23.5)	438 (22.5)
Bertagna et al. (2015)	2015	Italy	2005–2013	Patients underwent PET for oncologic purposes not related to breast disease	NR	42,927	NR	79 (0.18)
Shin et al. (2015)	2015	Korea	2005–2011	Patients underwent PET scans for cancer evaluation or health check-up	21,224		214 (1.01)	

*NR* not reported, *FIBU* focal incidental breast uptake

*Data was shown as frequency (percentage)

Within a total of 180,002 included scans, 109,947 scans (61%) were related to the patients who underwent FDG-PET/CT as part of staging of known or suspected primary malignancies, treatment follow-up and cancer screening (Shin et al. 2015; Bertagna et al. 2015; Chae et al. 2012; Kim et al. 2012; Lim et al. 2013), 8001 scans (4.4%) were related to the patients who underwent FDG-PET/CT for any reason except breast malignancy (Benveniste et al. 2015; Dunne et al. 2013), and the third group of patients with 62,054 scans (34.4%) were performed during a nationwide cancer screening program in Japan (Minamimoto et al. 2015).

### FIBU and risk of malignancy

The prevalence of FIBU was between 0.18 (Bertagna et al. 2015) and 22.5% (Benveniste et al. 2015) for both genders with a median of 0.52% (Kim et al. 2012) when considering the eight studies included in the systematic review. The prevalence of FIBU was between 0.51 (Minamimoto et al. 2015) and 23.5% (Benveniste et al. 2015) when considering female patients only, which was mentioned separately in five studies. The high reported prevalence by Benveniste et al. (Benveniste et al. 2015) was probably due to selection bias, since all included patients were staged for malignancy and the word “breast” was used in their search for the inclusion of patients from databases.

The prevalence of malignancy in patients undergoing further investigation of FIBU ranged from 27.3 (Lim et al. 2013) to 71.4% (Bertagna et al. 2015) with a median of 44.4% (Chae et al. 2012; Kim et al. 2012). The rate of primary breast cancer among patients with malignant lesions was between 15.6 (Benveniste et al. 2015) and 100% (Lim et al. 2013) (Table 2).

**Table 2:** Results of further investigation on included patients with detected focal incidental breast uptake*

Items	Lost to follow-up	Included FIBU**	Detected lesions***		Primary BC****
Studies			Benign	Malignant	
Chae et al. (2012)	60 (45.8)	71	39 (54.9)	32 (45.1)	27 (84.38)
Kim et al. (2012)	0 (0)	27	12 (44.4)	15 (55.6)	13 (86.67)
Dunne et al. (2013)	11 (28.9)	39	18 (43.6)	17 (43.6)	15 (88.24)
Lim et al. (2013)	10 (76.7)	33	24 (72.7)	9 (27.3)	9 (100)
Minamimoto et al. (2015)	NR	317	NR	135 (42.6)	123 (91.1)
Benveniste et al. (2015)	83 (18.9)	355	195 (54.9)	160 (45.1)	25 (15.63)
Bertagna et al. (2015)	44 (55.6)	35	10 (28.6)	25 (71.4)	23 (92.00)
Shin et al. (2015)	123 (57.4)	91	64 (70.3)	27 (29.7)	25 (92.59)

*Data was shown as frequency (percentage)

**Final included FIBU patients after lost to follow-up

***Classification to malignant and benign lesion were done based on histopathology results

****Frequency and percentage of primary breast cancer patients out of detected malignant lesions

*NR* not reported, *FIBU* focal incidental breast uptake, *BC* breast cancer

The histopathology of the detected lesions among further investigated FIBUs showed that the most common diagnosis for malignant and benign lesions was invasive ductal carcinoma (68.7%) and fibro-adenoma (37.6%), respectively. The median prevalence of primary breast cancer among the malignant breast tumors was 88.2%. Histopathological diagnosis of breast cancer is summarized in Table 3.

**Table 3:** Histopathological characteristics of patients with breast cancer*

Histopathology	Primary BC					Metastatic BC
Studies	IDC	DCIS	ILC	PC	Others**	
Chae et al. (2012)	23 (71.8)	3 (9.3)	1 (3.3)	–	–	5 (15.6)
Kim et al. (2012)	10 (66.6)	3 (20)	–	–	–	2 (13.3)
Dunne et al. (2013)	8 (47)	2 (11.7)	1 (5.8)	1 (5.8)	3 (17.6)	2 (11.7)
Lim et al. (2013)	5 (55.5)	3 (33.3)	–	1 (11.1)	–	–
Minamimoto et al. (2015)	95 (77.2)	27 (21.9)	–	–	1 (0.8)	–
Benveniste et al. (2015)	19 (11.8)	1 (0.6)	1 (0.6)	2 (1.2)	2 (1.2)	135 (84.3)
Bertagna et al. (2015)	17 (68)	2 (8)	4 (16)	–	–	2 (8)
Shin et al. (2015)	21 (77.7)	1 (3.7)	1 (3.7)	–	2 (7.4)	2 (7.4)

*Data was shown as frequency (percentage)

**Others consisted of invasive mixed mucinous and ductal carcinoma, metaplastic breast cancer, papillary lesion with atypia, medullary carcinoma, tubular adenocarcinoma, sarcomatiod tumor, and fibrocarcinoma

*IDC* invasive ductal carcinoma, *DCIS* ductal carcinoma in situ, *ILC* invasive lobular carcinoma, *PC* papillary carcinoma, *BC* breast cancer, *FIBU* focal incidental breast uptake

### Meta-analysis

Critical appraisal was achieved for eight studies regarding inclusion to the meta-analysis, and three (Bertagna et al. 2015; Chae et al. 2012; Kim et al. 2012) were excluded due to the absence of separate data for women (81,129 scans). Also, the study of Benveniste et al. (Benveniste et al. 2015) was excluded due to significant selection bias (1951 scans). Therefore, 83,080 scans were excluded due to absence of data regarding patients’ gender or selection bias, and the meta-analysis was finally performed on data from four studies (Shin et al. 2015; Minamimoto et al. 2015; Dunne et al. 2013; Lim et al. 2013). Included data comprised 93,591 women with FDG-PET/CT scans of which 480 were found to have a FIBU.

**Fig. 3:**
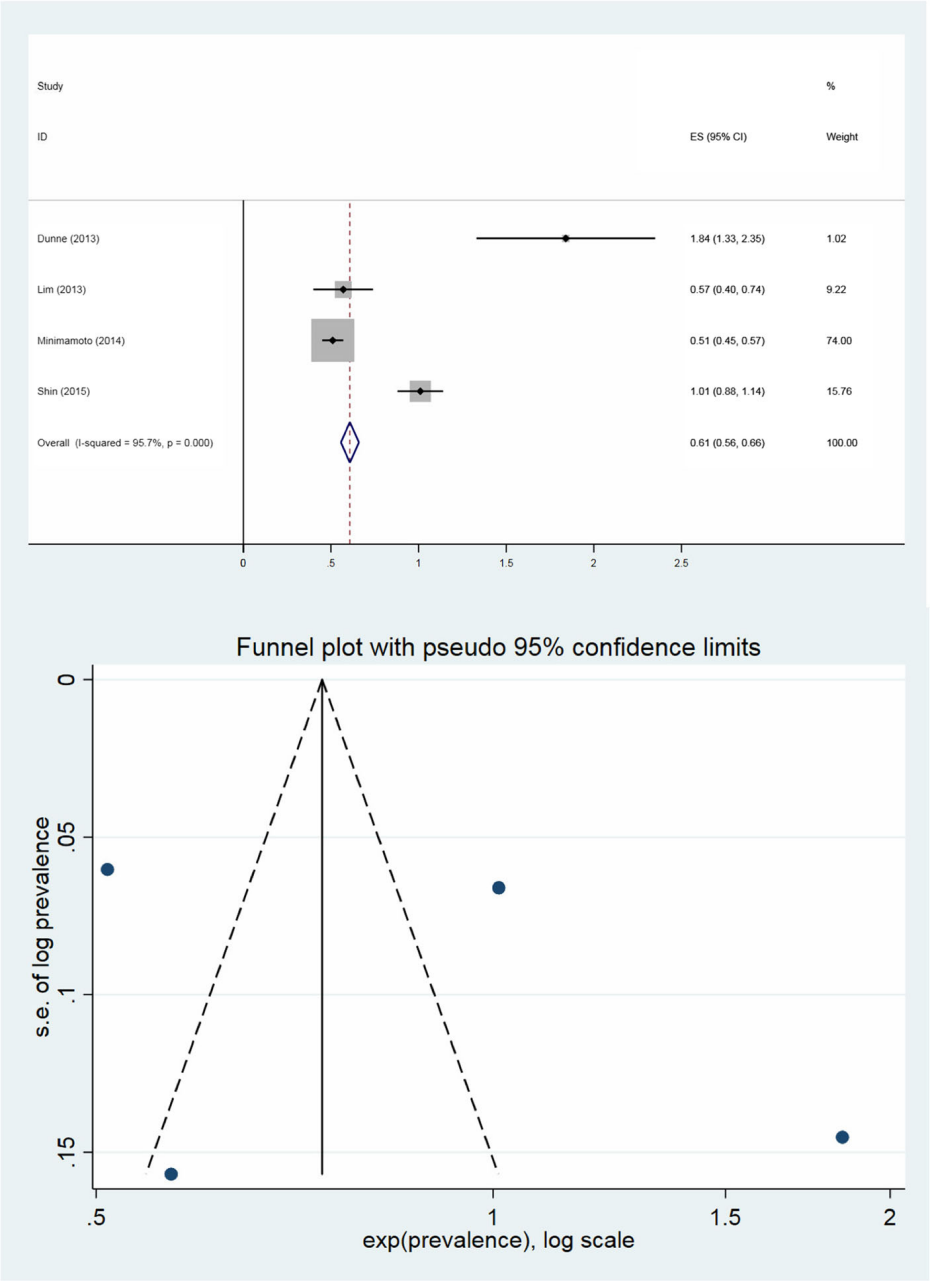
Pooled prevalence of focal incidental breast uptake in female patients

**Fig. 4:**
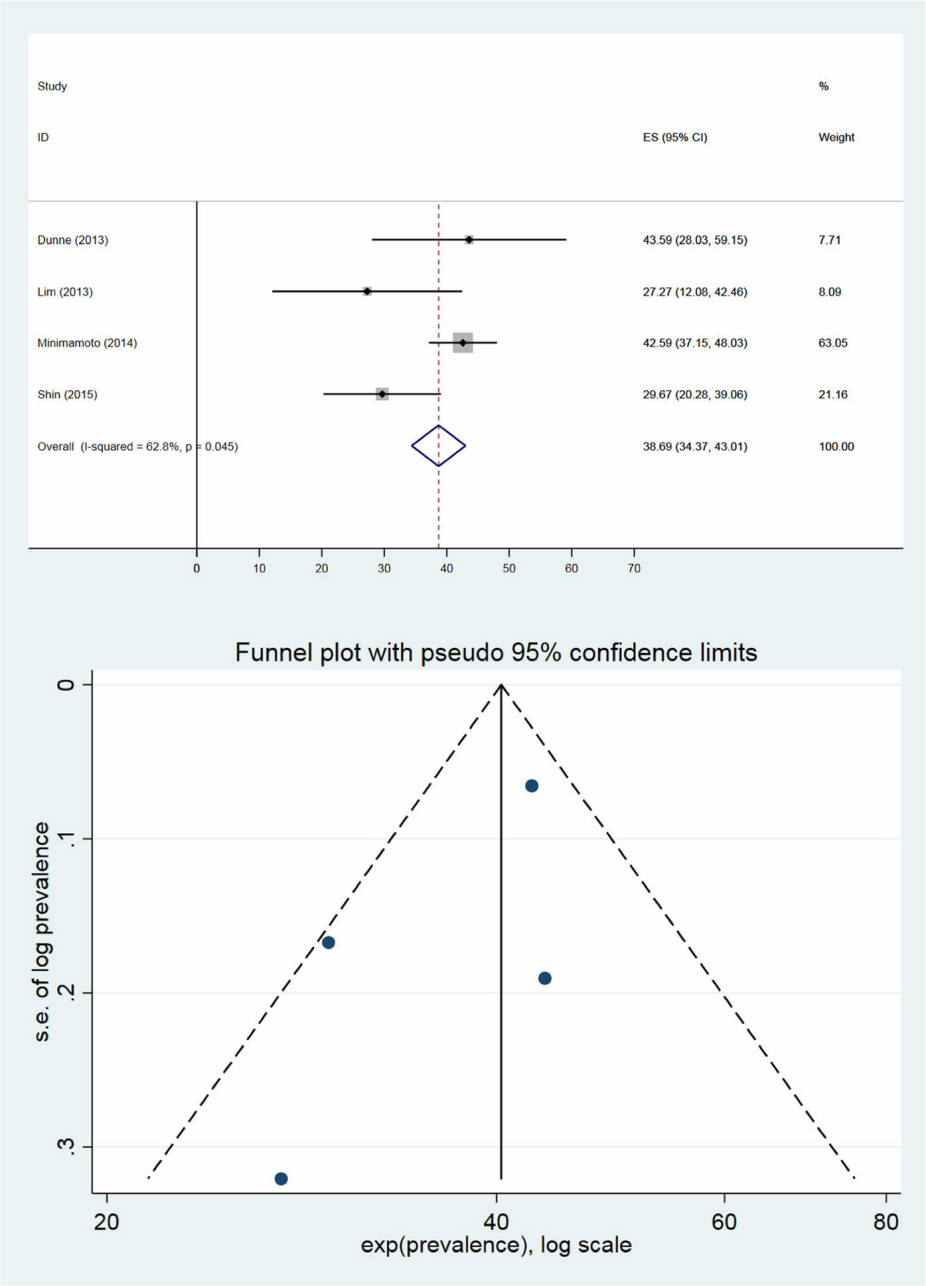
Pooled prevalence of further investigated malignancy in patients with focal incidental breast uptake

The median proportion of FIBU in women was 0.79% (range 0.51–1.84%) and the median proportion of malignancy in patients with FIBU was 36.2% (range 27.3–43.6%). The pooled prevalence of FIBU in female patients was 0.61% (95% CI; 0.56–0.66%), which has been shown in Fig. 3. *I*^2^ was 95.7% suggesting a high degree of heterogeneity between the studies, where only one of the four studies was within the funnel plot. This may indicate publication bias. The pooled prevalence of malignancy for FIBUs was 38.7% (95% CI 34.4–43.0%, range 27.3–43.6%) with lower heterogeneity (*I*^2^ = 62.8%). The associated funnel plot comprised all four studies within the funnel (Fig. 4).

## Discussion

Results on the prevalence of FIBU on FDG-PET/CT and the associated risk of malignancy were reported in this systematic review including eight studies, four of which entered the meta-analysis. We found a very low prevalence of FIBU (around 1%) for women undergoing FDG-PET/CT, but the risk of malignancy among them was considerable (about one third). The most frequently detected malignancy was invasive ductal carcinoma, which was found in about two thirds of all detected breast cancer patients.

The importance of further investigations and follow-up for this small group of patients is considered remarkable due to the high risk of malignancy in detected FIBU lesions (38%) including mostly primary breast cancer (over 80%). Further investigation of FIBU could therefore contribute to improved survival rate by early diagnosis in initial stages. Therefore, we should use the valuable information regarding FIBU offered by performed FDG-PET/CT for any other reasons.

All of the included studies had a retrospective design, and most of them were performed using database search for patients with FDG-PET/CT evaluation within a specific time-frame. There was a significant loss to follow-up in several studies except for the largest one (Minamimoto et al. 2015), which was performed in a screening setting. This study (Minamimoto et al. 2015) had the major impact on the result of our meta-analysis due to the huge sample size, which heavily influenced the pooled prevalence found in the current review.

There was a wide spectrum of inclusion criteria in performed studies, which were patients undergoing FDG-PET/CT for staging or follow-up of their primary malignancy (Bertagna et al. 2015), asymptomatic females (Minamimoto et al. 2015), and mix group of patients (Shin et al. 2015; Lim et al. 2013). These different populations under study may explain the large variance in rates of malignancies (27% up to 71%) in patients with detected FIBUs.

Also, bias related to timing and period of follow-up may have affected the results. More than 30% were lost to follow-up in most of the studies (Shin et al. 2015; Bertagna et al. 2015; Chae et al. 2012; Lim et al. 2013), which was partly due to an insufficient follow-up period (most studies had a minimum of 2 years for follow-up period as one of their exclusion criteria), and partly, it was due to disseminated primary malignancy and poor clinical condition that made further investigation irrelevant for the subjects. This might have caused an underestimation of patients with potentially malignant histological findings. It is worth mentioning that updates of protocols followed by increasing use of FDG-PET/CT could have affected our results due to the application of different PET/CT techniques over time.

A previous systematic review and meta-analysis about prevalence and clinical significance of incidental FDG breast uptake were conducted in 2013 by Bertagna et al. (2014). They selected 20 articles for their systematic review and found 13 articles eligible for meta-analysis; included articles were published until July 2013. Two of the studies included in our study (Dunne et al. 2013; Lim et al. 2013) were included in their report as well. They reported a pooled prevalence of FIBU for both genders and female patients to be 0.40% and 0.82%, respectively. Their reported pooled risk of malignancy among patients with FIBU was 48% (range 18–100%), which was higher compared to the 38.7% (range 27.3–43.6%) reported in our study. Two main reasons could justify the higher rate of malignancy in the previous review. First, included patients in the previous study had a known underlying malignancy, while most of included patients in our study were asymptomatic patients without a known history of malignancy. Secondly, the majority of analyzed patients with FIBU in the previous review were from the USA (6 of 13 studies), while most studies in the current review included patients with FIBU (over 90%) from Japan (Minamimoto et al. 2015) and South Korea (Shin et al. 2015; Lim et al. 2013). This could affect the results, since it has already been known that the incidence of breast cancer is higher in the USA and western countries than in Japan and Eastern Asian countries (Global Burden of Disease Cancer C 2017). Therefore, it could be expected to have lower rates of breast cancer in our study comparing to the previous review. On the other hand, it should be declared that the pooled risk of malignancy among patients with FIBU in previous review was out of patients including both genders, while we have reported the rate of malignancy out of only female patients. Since, the male breast cancers representing approximately 1% of all breast cancer worldwide (Gucalp et al. 2018), considering both genders, may have caused an underestimation of the exact rate of further investigated malignancies among patients with FIBU.

Both reviews have high heterogeneity from diversity in methodological aspects between studies, which could affect the outcome. Also, the most frequently reported malignancy was infiltrating ductal carcinoma in both reviews.

The small number of studies included in the meta-analysis as well as the large heterogeneity are limitations of our study. Another limitation is the high bias regarding flow and timing, as many patients did not receive a reference standard. Another topic that could have been addressed is the SUV_max_ in relation to benign and malignant lesions, which has been mentioned in some of the studies (Shin et al. 2015; Chae et al. 2012; Kim et al. 2012; Dunne et al. 2013). A significant difference in SUV_max_ has been reported between benign and malignant lesions (Kang et al. 2011; Litmanovich et al. 2009). However, some studies were against the value of these findings due to overlap of confidence interval for mean SUV_max_ between benign and malign lesions and lack of clinical efficacy (Lim et al. 2013; Chung et al. 2010).

Advantages of the current study are the update on literature, including studies with up-to-date technologies and the focus on FIBU and malignancy rates in women. Due to the low rate of male breast cancer worldwide (Gucalp et al. 2018), including both genders in the population of study may cause an underestimation of the exact rate of FIBU and risk of malignancy.

## Conclusion

There is an ongoing advancement in technology and an increasing pattern in clinical utilization of FDG-PET/CT for oncological and non-oncological patients, which call for knowledge on how to tackle incidentally detected FDG uptake on scans. Based on the results of the current study, focal incidental FDG uptake in breast occurs rarely (0.79%) on FDG-PET/CT in female patients but reflects high probability of malignancy (36.2%). Therefore, focal incidental FDG uptake in breast should always be given appropriate consideration followed by further relevant clinical investigation.

## Additional file

Additional file 1: The search terms and overview of search protocol is presented in additional file 1. (PDF 980 kb)

## Data Availability

All data generated or analyzed during this study are included in this published article (and its supplementary information files).

## Abbreviations

FDG: Fluorine-18-fluorodeoxyglucose
FIBU: Focal incidental breast uptake
PET: Positron Emission Tomography
PET/CT: Positron emission tomography integrated with computed tomography
