# Supplementary material for: Prevalence of focal incidental breast uptake on FDG-PET/CT and risk of malignancy: a systematic review and meta-analysis
Source: Eur J Hybrid Imaging. 2019 Sep 30;3:16. doi: 10.1186/s41824-019-0063-5 (PMC8218088; doi:10.1186/s41824-019-0063-5)
Supplement: Supplementary file 1 — The search terms and overview of search protocol is presented in additional file 1. (PDF 980 kb) [file 41824_2019_63_MOESM1_ESM.pdf]

ovidsp.uk.ovid.com.proxy1-bib.sdu.dk:2048/sp-3.28.0a/ovidweb.cgi?S=DODCPDHFEFHFNEDGFNFKLHPFHGFLAA00&Main+Search+Page=1

Apper For rask tilgang plasserer du bokmerkene her i bokmerkeraden. Importér bokmerker nå Andre bokmerk

# ▲	Searches	Results	Type	Actions	Annotations
1	(positron emission tomograph* or PET* or petscan* or FDG-PET* or PET-FDG* or PETFDG* or FDGPET*).mp. [mp=title, abstract, heading word, drug trade name, original title, device manufacturer, drug manufacturer, device trade name, keyword, floating subheading word]	347872	Advanced	Display Results | More ▾	
2	((fluorodeoxyglucose adj1 F18) or (fluorodeoxyglucose adj1 F 18) or (fludeoxyglucose adj1 F18) or (fludeoxyglucose adj1 F 18) or fluorine-18-fluorodeoxyglucose or fluorine-18-fludeoxyglucose or (fluorodeoxyglucose adj1 18F) or (fluorodeoxyglucose adj1 18 F) or (fludeoxyglucose adj1 18F) or fludeoxyglucose 18 F or 18 FDG or 18FDG or 18 F FDG or 18F FDG or F18 FDG or F 18 FDG or FDG 18F or FDG 18 F or FDG F18 or FDG F 18 or FDG18 or 18 Fluorodeoxyglucose or 18Fluorodeoxyglucose or 2-Fluoro-d-deoxy-D-glucose or 2-Fluoro-2-deoxyglucose or 2-Fluoro-2-deoxy-glucose).mp. [mp=title, abstract, heading word, drug trade name, original title, device manufacturer, drug manufacturer, device trade name, keyword, floating subheading word]	57057	Advanced	Display Results | More ▾	
3	exp positron emission tomography/	126378	Advanced	Display Results | More ▾	
4	exp positron emission tomography-computed tomography/	9447	Advanced	Display Results | More ▾	
5	exp fluorodeoxyglucose f 18/	50039	Advanced	Display Results | More ▾	
6	1 or 2	352051	Advanced	Display Results | More ▾	
7	3 or 4 or 5	142864	Advanced	Display Results | More ▾	
8	6 or 7	352057	Advanced	Display Results | More ▾	
9	exp incidental finding/ or exp incidentaloma/	15795	Advanced	Display Results | More ▾	
10	unexpect*.mp.	133738	Advanced	Display Results | More ▾	
11	foc*.mp.	1223974	Advanced	Display Results | More ▾	
12	(added or adding or additional).mp. [mp=title, abstract, heading word, drug trade name, original title, device manufacturer, drug manufacturer, device trade name, keyword, floating subheading word]	1294671	Advanced	Display Results | More ▾	
13	exp incidental finding/ or finding*.mp.	2647999	Advanced	Display Results | More ▾	
14	uptake*.mp.	474756	Advanced	Display Results | More ▾	
15	coincident*.mp.	31767	Advanced	Display Results | More ▾	
16	9 or 10 or 11 or 12 or 13 or 14 or 15	5248677	Advanced	Display Results | More ▾	
17	exp breast tissue/ or exp breast/ or breast*.mp. or exp breast density/	711801	Advanced	Display Results | More ▾	
18	exp breast/ or mamma*.mp.	600008	Advanced	Display Results | More ▾	
19	exp mammary gland/ or mammary gland*.mp. or exp breast/	138561	Advanced	Display Results | More ▾	
20	17 or 18 or 19	1137277	Advanced	Display Results | More ▾	
21	8 and 16 and 20	6617	Advanced	Display Results | More ▾	
22	limit 21 to (books or "book review" or chapter or conference abstract or conference paper or "conference review")	2570	Advanced	Display Results | More ▾	
23	21 not 22	4047	Advanced	Display Results | More ▾	
24	23	4047	Advanced	Display Results | More ▾	
25	limit 24 to (human and english language and yr="2012 -Current")	1736	Advanced	Display Results | More ▾	

▼ Search History (23)

View Saved

# ▲	Searches	Results	Type	Actions	Annotations
1	(positron emission tomograph* or PET* or petscan* or FDG-PET* or PET-FDG* or PETFDG* or FDGPET*).mp. [mp=title, abstract, original title, name of substance word, subject heading word, keyword heading word, protocol supplementary concept word, rare disease supplementary concept word, unique identifier, synonyms]	197120	Advanced	Display Results | More ▾	
2	((fluorodeoxyglucose adj1 F18) or (fluorodeoxyglucose adj1 F 18) or (fludeoxyglucose adj1 F18) or (fludeoxyglucose adj1 F 18) or fluorine-18-fluorodeoxyglucose or fluorine-18-fludeoxyglucose or (fluorodeoxyglucose adj1 18F) or (fluorodeoxyglucose adj1 18 F) or (fludeoxyglucose adj1 18F) or fludeoxyglucose 18 F or 18 FDG or 18FDG or 18 F FDG or 18F FDG or F18 FDG or F 18 FDG or FDG 18F or FDG 18 F or FDG F18 or FDG F 18 or FDG18 or 18 Fluorodeoxyglucose or 18Fluorodeoxyglucose or 2-Fluoro-d-deoxy-D-glucose or 2-Fluoro-2-deoxyglucose or 2-Fluoro-2-deoxy-glucose).mp. [mp=title, abstract, original title, name of substance word, subject heading word, keyword heading word, protocol supplementary concept word, rare disease supplementary concept word, unique identifier, synonyms]	32901	Advanced	Display Results | More ▾	
3	positron emission tomography.mp. or exp Positron-Emission Tomography/	76109	Advanced	Display Results | More ▾	
4	positron emission tomography-computed tomography.mp. or exp Positron Emission Tomography Computed Tomography/	8290	Advanced	Display Results | More ▾	
5	exp FLUORODEOXYGLUCOSE F18/ or fluorodeoxyglucose.mp.	30719	Advanced	Display Results | More ▾	
6	1 or 2	198875	Advanced	Display Results | More ▾	
7	3 or 4 or 5	80292	Advanced	Display Results | More ▾	
8	6 or 7	198950	Advanced	Display Results | More ▾	
9	incident*.mp.	122536	Advanced	Display Results | More ▾	
10	unexpect*.mp.	104914	Advanced	Display Results | More ▾	
11	foc*.mp.	951295	Advanced	Display Results | More ▾	
12	(added or adding or additional).mp. [mp=title, abstract, original title, name of substance word, subject heading word, keyword heading word, protocol supplementary concept word, rare disease supplementary concept word, unique identifier, synonyms]	948090	Advanced	Display Results | More ▾	
13	finding*.mp. or exp INCIDENTAL FINDINGS/	2003493	Advanced	Display Results | More ▾	
14	uptake*.mp.	343878	Advanced	Display Results | More ▾	
15	coincidental*.mp.	6709	Advanced	Display Results | More ▾	
16	9 or 10 or 11 or 12 or 13 or 14 or 15	4042654	Advanced	Display Results | More ▾	
17	exp BREAST/ or exp BREAST DENSITY/ or breast*.mp.	453424	Advanced	Display Results | More ▾	
18	mamma*.mp. or exp Breast/	481990	Advanced	Display Results | More ▾	
19	exp Mammary Glands, Animal/ or mammary gland*.mp.	32183	Advanced	Display Results | More ▾	
20	17 or 18 or 19	851339	Advanced	Display Results | More ▾	
21	8 and 16 and 20	2910	Advanced	Display Results | More ▾	
22	21	2910	Advanced	Display Results | More ▾	
23	limit 22 to (english language and humans and yr="2012 -Current")	914	Advanced	Display Results | More ▾	

pet incident og breast blok

To search an exact word(s) use quotation marks, e.g. "hospital" finds hospital; hospital (no quotation marks) finds hospital and hospitals; pay finds paid, pays, paying, payed)

Add to top

View fewer lines

#1	(positron emission tomograph* or PET* or petscan* or FDG-PET* or PET-FDG* or PETFDG* or FDGPET*)	34057
#2	((fluorodeoxyglucose adj1 F18) or (fluorodeoxyglucose adj1 F 18) or (fludeoxyglucose adj1 F18) or (fludeoxyglucose adj1 F 18) or fluorine-18-fluorodeoxyglucose or fluorine-18-fludeoxyglucose or (fluorodeoxyglucose adj1 18F) or (fluorodeoxyglucose adj1 18 F) or (fludeoxyglucose adj1 18F) or fludeoxyglucose 18 F or 18 FDG or 18FDG or 18 F FDG or 18F FDG or F18 FDG or F 18 FDG or FDG 18F or FDG 18 F or FDG F18 or FDG F 18 or FDG18 or 18 Fluorodeoxyglucose or 18Fluorodeoxyglucose or 2-Fluoro-d-deoxy-D-glucose or 2-Fluoro-2-deoxyglucose or 2-Fluoro-2-deoxy-glucose)	1817
#3	MeSH descriptor: [Positron-Emission Tomography] explode all trees	1332
#4	positron emission tomography- computed tomography	1755
#5	MeSH descriptor: [Fluorodeoxyglucose F18] explode all trees	833
#6	#1 or #2 or #3 or #4 or #5	34111
#7	MeSH descriptor: [Incidental Findings] explode all trees	38
#8	(incident* or unexpect* or foc* or added og additional or adding or finding* or uptake* or coincident*)	167829
#9	#7 or #8	167829
#10	MeSH descriptor: [Breast] explode all trees	741
#11	MeSH descriptor: [Mammary Glands, Human] explode all trees	36
#12	(breast* or mamma* or mammary gland*)	39076
#13	#10 or #11	741
#14	#13 or #12	39082
#15	#6 and #9 and #14	1435
#16	#15<br>Online Publication Date from Jan 2012 to Mar 2018	829
